# Transcriptome Study in Sicilian Patients with Autism Spectrum Disorder

**DOI:** 10.3390/biomedicines12071402

**Published:** 2024-06-25

**Authors:** Michele Salemi, Francesca A. Schillaci, Giuseppe Lanza, Giovanna Marchese, Maria Grazia Salluzzo, Angela Cordella, Salvatore Caniglia, Maria Grazia Bruccheri, Anna Truda, Donatella Greco, Raffaele Ferri, Corrado Romano

**Affiliations:** 1 Oasi Research Institute—IRCCS, 94018 Troina, Italy; fran7.sch@gmail.com (F.A.S.); glanza@oasi.en.it (G.L.); msalluzzo@oasi.en.it (M.G.S.); scaniglia@oasi.en.it (S.C.); mariagraziabruccheri@gmail.com (M.G.B.); dgreco@oasi.en.it (D.G.); rferri@oasi.en.it (R.F.); cromano@oasi.en.it (C.R.); 2 Department of Surgery and Medical—Surgical Specialties, University of Catania, 95124 Catania, Italy; 3 Genomix4Life S.r.l., 84081 Baronissi, Italy; giovanna.marchese@genomix4life.com (G.M.); angela.cordella@genomix4life.com (A.C.); anna.truda@genomix4life.com (A.T.); 4 Genome Research Center for Health—CRGS, 84081 Baronissi, Italy; 5 Department of Biomedical and Biotechnological Sciences, University of Catania, 95124 Catania, Italy

**Keywords:** mRNAs, RNA sequencing, autism spectrum disorder, transcriptome analysis

## Abstract

ASD is a complex condition primarily rooted in genetics, although influenced by environmental, prenatal, and perinatal risk factors, ultimately leading to genetic and epigenetic alterations. These mechanisms may manifest as inflammatory, oxidative stress, hypoxic, or ischemic damage. To elucidate potential variances in gene expression in ASD, a transcriptome analysis of peripheral blood mononuclear cells was conducted via RNA-seq on 12 ASD patients and 13 healthy controls, all of Sicilian ancestry to minimize environmental confounds. A total of 733 different statistically significant genes were identified between the two cohorts. Gene Set Enrichment Analysis (GSEA) and Gene Ontology (GO) terms were employed to explore the pathways influenced by differentially expressed mRNAs. GSEA revealed GO pathways strongly associated with ASD, namely the GO Biological Process term “Response to Oxygen-Containing Compound”. Additionally, the GO Cellular Component pathway “Mitochondrion” stood out among other pathways, with differentially expressed genes predominantly affiliated with this specific pathway, implicating the involvement of different mitochondrial functions in ASD. Among the differentially expressed genes, *FPR2* was particularly highlighted, belonging to three GO pathways. *FPR2* can modulate pro-inflammatory responses, with its intracellular cascades triggering the activation of several kinases, thus suggesting its potential utility as a biomarker of pro-inflammatory processes in ASD.

## 1. Introduction

Dysfunction in communication and social interaction, sensory impairments, restricted interests, repetitive behaviors, and stereotypies, alongside varying degrees of intellectual disability, constitute the cardinal manifestations of Pervasive Developmental Disorders (PDDs) [1,2]. This term was later replaced by “autism spectrum disorder” (ASD), as defined in the 11th edition of the International Classification of Diseases (ICD-11) and the fifth edition of the Diagnostic and Statistical Manual of Mental Disorders (DSM-5), due to the blurred boundaries between the various pervasive developmental disorders (PDDs) [1,2]. ASD encompasses autistic disorder, Asperger’s disorder, and pervasive developmental disorder not otherwise specified (PDD-NOS) [2].

Autism is classified as an early neurodevelopmental disorder, characterized by high heterogeneity and a heritability estimated between 40% and 90% [1]. Beyond the aforementioned characteristics, individuals with autism may also experience comorbid conditions such as anxiety, depression, attention-deficit/hyperactivity disorder (ADHD), sleep disturbances, epilepsy, gastrointestinal and immunological dysfunctions, feeding difficulties, and various medical comorbidities (e.g., fragile X syndrome, Down syndrome, Duchenne muscular dystrophy, neurofibromatosis type I, mitochondrial disorders, disorders of creatine metabolism, and endocrine disorders) [3,4].

The prevalence of ASD has significantly risen over time, attributed not only to increased awareness and expanded diagnostic criteria but also to enhanced healthcare services [2,5]. Various theories, ranging from being raised by wolves (the “wild boy of Aveyron”) to genetic predispositions, have been proposed and refuted regarding the etiology of ASD [1,2]. Presently, ASD is recognized as a multifactorial condition with a primary genetic basis, modulated by environmental risk factors that can influence genetic and epigenetic mechanisms, as well as induce inflammatory, oxidative stress, hypoxic, or ischemic damage [1,2]. Additionally, prenatal and perinatal factors, including maternal lifestyle and diet, advanced parental age, maternal obesity, hypertension, infections, birth complications, gestational diabetes, short inter-pregnancy intervals, exposure to valproic acid, premature rupture of membranes, and assisted reproductive technologies, are acknowledged contributors to ASD development [1,3,5,6]. It remains challenging to ascertain whether these associations are causative or contributory to ASD onset [1,3]. Twin and familial studies estimate ASD heritability between 0.69 and 0.91, indicating polygenic risk involvement with variable contributions from de novo or rare variants and the cumulative effects of common variants [7,8]. ASD phenotypes result from intricate interactions among environmental and genetic factors, epigenetic modifications, and fetal brain developmental events [9].

Recent advancements in investigative techniques, applied in both neurodevelopmental and neurodegenerative disorders, have revealed gene enrichments in ASD associated with transcription, translation, synaptic function, epigenetics, immunity, and inflammation [10,11]. Recently, using the GO database, association enrichment analysis of pathways or groups of genes has gained prominence, as the disruption of an entire biological pathway is thought to be more implicated in disease genesis due to genetic polymorphisms functionally related to the same pathway. Additionally, genetic heterogeneity suggests that multiple genes may be involved in the same biological pathway, so assessing the pathway may increase the ability to detect associations between genes and disease [12,13]. Meta-analyses of transcriptomic data from peripheral blood samples in ASD subjects have shown transcriptional alterations in the innate immune system and inflammatory signaling pathways [14]. Studies have identified differentially expressed genes such as High Mobility Group Box 3 (*HMGB3*), Protein Tyrosine Phosphatase, Receptor-Type, N, Polypeptide 2 (*PTPRN2*), and Neuromedin U Receptor 1 (*NMUR1*) [15]. Comparative gene expression profiling studies have identified differentially expressed genes and significant KEGG signaling pathways associated with ASD [16,17].

In our study, we used peripheral blood mononuclear cell (PBMCs) samples as they are easily accessible, whereas ASD-related brain studies are very difficult and are performed at a limited level [18]. In addition, Sullivan et al. [19] found a shared expression profile between different central nervous system tissues and blood, suggesting the use of peripheral blood expression as a surrogate for the brain [19,20]. Others, such as Glatt et al. (2012), have performed an analysis of PBMCs developing a panel of early biomarkers of risk for autism in infants and young children [18,19,21,22]. In relation to the aforementioned bibliographic data, which emphasize the importance of gene expression in patients with ASD, we aimed to evaluate the transcriptome in a cohort of Sicilian patients with ASD compared to a cohort of healthy controls. This was achieved using a latest-generation Illumina sequencing platform.

## 2. Materials and Methods

### 2.1. Patients and Controls Selection

A transcriptome analysis of PBMCs was conducted using RNA-seq in a cohort of 25 subjects, comprising 12 ASD patients (10 males and 2 females, median and range for patient age 17.58 ± 12.88 years) and 13 healthy controls (CTRL) (11 males and 2 females, median and range for patient age 19.15 ± 12.07 years). All participants were recruited from the Oasi Research Institute-IRCCS, Troina (Italy) and originated from the same region (Sicily) to minimize environmental influences. Diagnoses of ASD were made by a child neuropsychiatrist and a pediatric neurologist according to current DSM-V criteria [23]. Confirmation of diagnosis was obtained through neuropsychological and psychopathological evaluations conducted by trained developmental psychologists. Additionally, the Autism Diagnostic Observation Schedule—Generic (ADOS-G) or its revised version (ADOS-2) was utilized [24,25].

All participants, except for four ASD patients, had normal cognitive status. Among the ASD patients, two had moderate intellectual disability, and two had borderline intellectual disability. Language impairment was observed in four ASD children, developmental coordination disorder in two, ADHD in two, and motor and vocal tic disorder in one. Electroencephalography results were normal in all patients, except for two: one exhibited clear epileptiform discharges, while the other showed sporadic non-specific alterations. Although among the patients only three had taken melatonin 2 mg/day, they had discontinued it 10 days before the blood sample was taken.

The healthy controls were free of medication, had no history of neurological or psychiatric disorders, and underwent entirely normal neurological examinations. Informed consent was obtained from all participants or their guardians, and the study was conducted in accordance with the Declaration of Helsinki of 1964 and its later amendments. Approval was obtained from the Ethics Committee of the Oasi Research Institute—IRCCS, Troina (Italy) (approval number 2022/04/05/CE-IRCCS-OASI/52). All participants or their guardians provided informed consent for publication. 

### 2.2. RNA Extraction Subsection

PBMCs were isolated using Ficoll-Paque (Ficoll Plaque PLUS-GE Healthcare Life Sciences, Piscataway, NJ, USA), followed by RNA extraction using TRIzol reagent (Invitrogen Life Technologies, Carlsbad, CA, USA) according to the manufacturer’s instructions [10]. The extracted RNA was stored at −80 °C until further processing. RNA concentration and purity were assessed using the NanoDropOne (Thermo Fisher Scientific, Waltham, MA, USA), and sample integrity was evaluated with the Tape Station 4200 (Agilent Technologies, Santa Clara, CA, USA) using an RNA screen tape assay.

### 2.3. RNA Sequencing and Data Analysis 

RNA sequencing and subsequent data analysis were conducted by Genomix4Life Srl (Baronissi, Italy). Indexed libraries were prepared from 800 ng purified RNA using the Illumina^®^ Stranded mRNA Prep Kit (Illumina, San Diego, CA, USA) following the manufacturer’s protocol. After mRNA enrichment and fragmentation, cDNA synthesis, adapter ligation, and PCR amplification were performed. Library quantification was carried out using the TapeStation 4200 (Agilent Technologies) and Qubit 4 fluorometer (Thermo Fisher Scientific, Waltham, MA, USA). Equimolar amounts of indexed libraries were pooled and sequenced on the Illumina NovaSeq6000 platform in a 2 × 75 paired-end format.

Raw sequence files (.fastq files) underwent quality control analysis using FastQC (http://www.bioinformatics.babraham.ac.uk/projects/fastqc; accessed on 1 January 2024). Low-quality reads, short reads (≤25 bp), and adaptor sequences were trimmed using Cutadapt (v.2.8) [26]. Subsequently, the fastq files were aligned to the reference genome using the bioinformatics tool STAR (version 2.7.3a) [27], employing default parameters for paired reads. The human assembly from GenCode (HG38 release 37 (GRCh38.p13)) was used as the reference genome. Quantification of expressed genes for each sample was computed using the featureCounts algorithm [28]. R was utilized to normalize the data using negative binomial generalized linear models, considering all genes expressed in ≥25% of samples, through the Bioconductor DESeq2 package. This model incorporates both the mean expression level and the dispersion parameter for each gene, allowing for robust estimation of expression differences between conditions. The Bioconductor DESeq2 package employs the Benjamini–Hochberg (BH) correction for padj calculation with default parameters. The BH correction is a method to control the false discovery rate (FDR), which is the expected proportion of false positives among the declared significant results [29]. Genes with a fold change ≥ 2.50 or ≤ −2.50 (|FC| ≥ 2.50) and adjusted *p* values ≤ 0.05 (padj) (pdaj ≤ 0.05, as suggested by the literature [29,30,31,32], were considered differentially expressed. Heatmaps and volcano plots of the differentially expressed genes were generated using the ComplexHeatmap version 2.14.0 [33] and ggplot2 version 3.5.1 [34] packages in R, respectively.

### 2.4. Functional and Pathways Analysis of Differentially Expressed Genes

To explore the pathways influenced by differentially expressed mRNAs (DEmRNAs) and identify significant modulations in autistic patients, we conducted a Gene Set Enrichment Analysis (GSEA) to determine whether predefined gene sets exhibit statistically significant, coherent differences between two biological states. We analyzed differentially expressed genes by calculating the enrichment score of specified gene sets across various functional categories to detect significant and coherent differences between the two conditions. Specifically, we selected the Gene Ontology C5 module within MSigDB to query Molecular Function (MF), Biological Process (BP), and Cellular Component (CC) ontologies.

A functional analysis of statistically significant genes was conducted using GSEA (v.4.1.0) [35], a computational method that evaluates RNA-Seq data at the gene set level. GSEA identifies whether predefined sets of genes show statistically significant, concordant differences between two biological states. The gene sets used for this analysis were obtained from the Molecular Signature Database (MSigDB) and consisted of genes listed by the HGNC gene symbol, ensuring standardized and universally recognized nomenclature. This approach allows for a more comprehensive understanding of the underlying biological processes, pathways, and functional categories associated with the conditions studied. The analysis provides insights into key biological mechanisms and highlights the most relevant gene sets that contribute to the observed differences in gene expression.

Cytoscape version 3.9.1 [36] was utilized for the visualization of selected Gene Ontology (GO) terms, enabling the depiction of complex networks. This software facilitates the construction and visualization of networks where nodes represent genes or gene products, and edges represent the biological relationships between them. The GO terms, which describe gene functions in categories such as biological processes, cellular components, and molecular functions, were mapped onto the network to highlight key interactions and pathways. Cytoscape’s advanced layout algorithms and visualization tools were used to optimize the representation, making it easier to interpret the interconnections and biological significance of the genes involved.

## 3. Results

### 3.1. Whole Transcriptome of Autistic Patients

**Figure 1:**
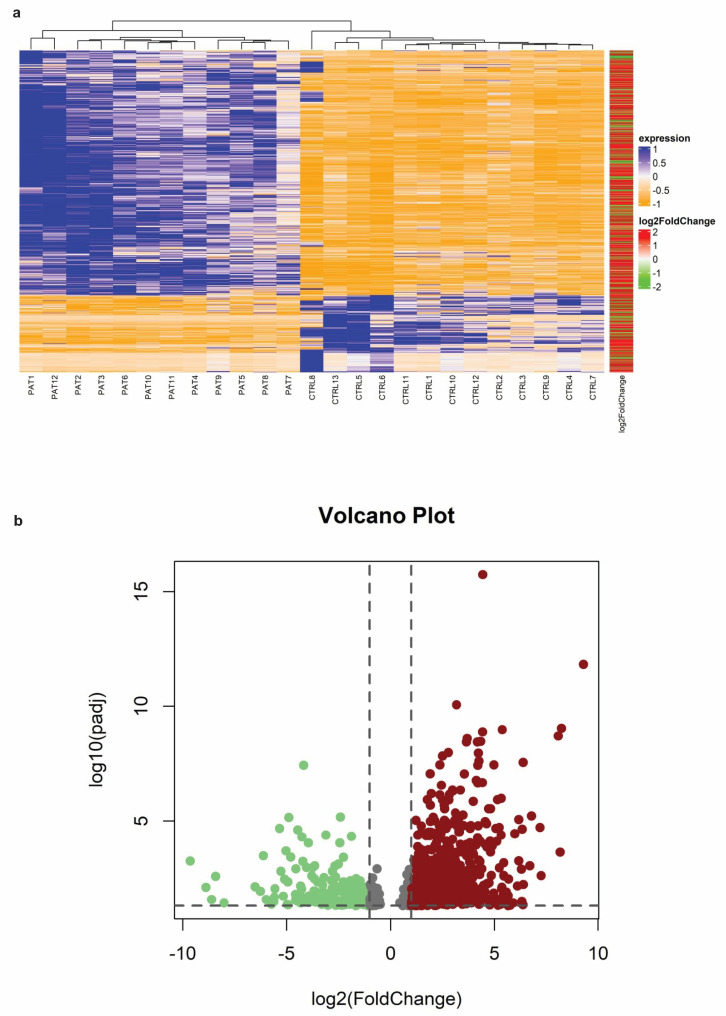
Differentially expressed genes (DEGs). (**a**) Heatmap illustrating significant DEGs in patients with ASD (PAT) compared to CTRL individuals. Genes with increased expression levels are depicted in orange, while those with decreased expression levels are shown in blue. The log2 (foldChange) bar indicates up-regulated genes in red and down-regulated genes in green; (**b**) volcano plot representing significant DEGs based on fold changes and *p*-values. Down-regulated genes are depicted in green, while up-regulated genes are shown in red. Up/down-regulated in ASD.

Whole-transcriptome RNA sequencing was conducted using next-generation sequencing in patients and controls with the aim of elucidating potential differences in gene expression between the two groups. Principal component analysis (PCA) was performed, revealing significant variance between the groups and complete separation between sample conditions. Gene expression levels were quantified using featureCounts with common gene annotations, identifying a total of 24,074 expressed genes across all samples. Hierarchical clustering analysis was employed to investigate overall gene expression differences, demonstrating consistent trends in gene expression changes between the two groups, with statistically significant differences observed (Figure 1a). Differential expression analysis identified 733 genes as statistically significant (padj ≤ 0.05) between the two groups (autistic vs. control) (Supplementary Table S1). Among these, 584 genes were significantly up-regulated (padj ≤ 0.05 and FC ≥ 2.5), while 149 genes were significantly downregulated (padj ≤ 0.05 and FC ≤ −2.5). Volcano plots depicted distinct differences in gene regulation between the treated and control groups (Figure 1b) (Supplementary Table S1). Raw data and normalized gene counts are available at ArrayExpress under accession number E-MTAB-13871.

### 3.2. The Gene Set Enrichment Analysis (GSEA)

Our dataset contained 733 native features, which were used for analysis. Gene set size filters (min = 15, max = 500) were applied, resulting in the filtering out of 15,641 out of 16,008 gene sets, as per GSEA default settings, and the remaining 367 gene sets were used in the analysis.

The analysis report comprises two “Enrichment in Phenotype” sections: the first section presents results for gene sets with a positive enrichment score (indicating enrichment at the top of the ranked list), while the second section displays results for gene sets with a negative enrichment score (indicating enrichment at the bottom of the ranked list) (Supplementary Tables S2 and S3).

In detail, among the 367 gene sets analyzed, 74 were up-regulated in the “na_pos” phenotype (positive enrichment score). Similarly, 293 gene sets were up-regulated in the “na_neg” phenotype (negative enrichment score), with 136 gene sets significantly enriched at FDR < 25%, 73 gene sets significantly enriched at nominal *p*-value < 0.01%, and 111 gene sets significantly enriched at nominal *p*-value < 0.05%.

**Figure 2:**
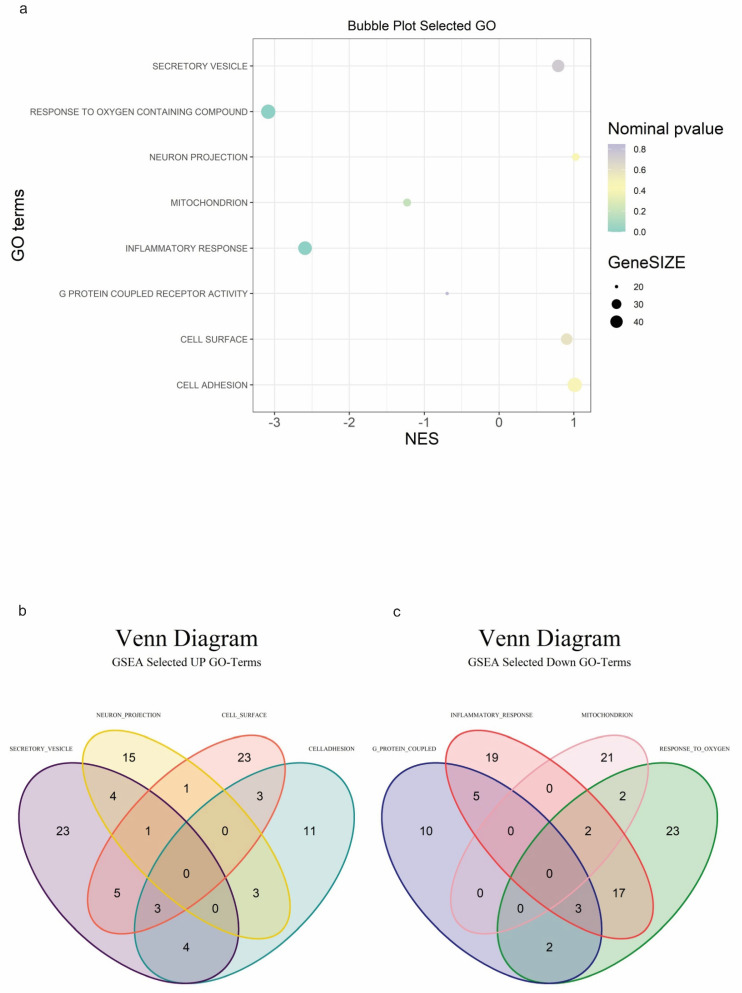
Gene Set Enrichment Analysis (GSEA) plots. (**a**) Bubble plot depicting selected Gene Ontology (GO) terms enriched in GSEA. Each GO term is represented by three distinct numerical parameters: Normalized Enrichment Score (NES), nominal *p*-value, and gene set size. In light green, more significant nominal *p*-values are indicated; (**b**) Venn diagram illustrating specific and common up-regulated transcripts among four selected GO terms; (**c**) Venn diagram demonstrating specific and common down-regulated transcripts among four selected GO terms.

Among all Gene Ontology (GO) terms (Figure 2a), we focused our attention on four GO terms up-regulated in the “na_neg” phenotype:

- “GOBP_Response_To_Oxygen_Containing_Compound” (Normalized Enriched Score (NES) −3.14), which includes processes such as oxidative stress response, oxygen transport, and cellular respiration.
- “GOBP_Inflammatory_Response” (NES −2.56), representing a set of genes known to be involved in different aspects of the inflammatory response. These genes may encode proteins involved in immune cell activation, cytokine production, chemotaxis, tissue remodeling, and other processes associated with inflammation.
- “GOCC_Mitochondrion” (NES −1.23), categorizing genes based on their subcellular localization within the mitochondrion. Genes categorized under this term typically encode proteins that are either integral or associated with the mitochondria, involved in various functions such as oxidative phosphorylation, metabolism, calcium signaling, and apoptosis.
- “GOMF_G_Protein_Coupled_Receptor_Activity” (NES −0.73), representing G protein-coupled receptors (GPCRs), a large family of cell surface receptors involved in transmitting signals from the external environment to the interior of the cell. They play crucial roles in a wide range of physiological processes, including sensory perception, neurotransmission, hormone regulation, and immune response. These GO terms share several genes in common with each other, as shown in Figure 2b.

Conversely, we also studied four GO terms in the “na_pos” phenotype, including GOCC_Neuron_Projection, GOBP_Cell_Adhesion, GOCC_Cell_Surface, and GOCC_Secretory_Vesicle.

- GOCC_Neuron_Projection (NES 1.02) includes genes involved in processes such as communication between neurons and signal transmission in the nervous system.
- GOBP_Cell_Adhesion (NES 1.01) is associated with processes by which cells interact and attach to neighboring cells or the extracellular matrix. Cell adhesion is essential for various biological processes, including tissue development, immune response, and wound healing.
- GOCC_Cell_Surface (NES 0.90) is involved in various functions such as cell signaling, adhesion, and interaction with other cells or molecules.
- GOCC_Secretory_Vesicle (NES 0.79) refers to secretory vesicles, membrane-bound vesicles within cells storing and transporting molecules for secretion. Secretory vesicles play a key role in exocytosis, the process by which cells release substances like hormones, neurotransmitters, or digestive enzymes into the extracellular space (Figure 2c).

Cytoscape analysis was conducted to further elucidate the connections between the selected Gene Ontology (GO) terms and the genes exhibiting differential expression across various categories of biological processes, molecular functions, or cellular components.

**Figure 3:**
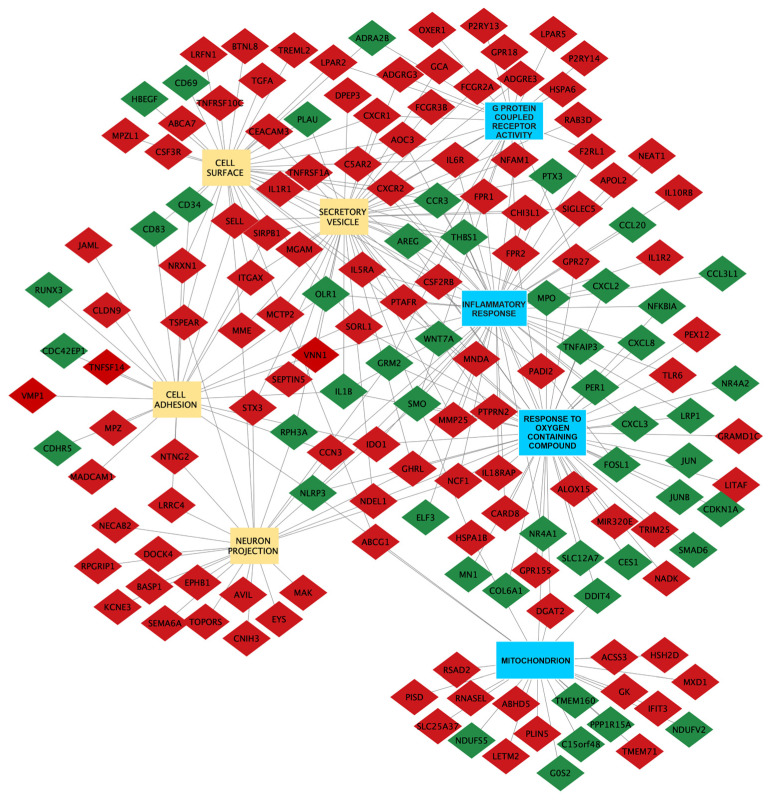
The network constructed using Cytoscape illustrates the relationship between dysregulated genes and GSEA pathways. Nodes in the network represent the Gene Ontology (GO) terms: yellow nodes indicate positive Normalized Enrichment Scores (NES), while blue nodes indicate negative NES. The green color denotes down-regulated genes, while the red color indicates up-regulated genes.

As depicted in Figure 3, each GO term is represented as a node, with genes linked to them as edges. By integrating gene expression data with GO term annotations and representing them as a network, we can highlight functional relationships between genes and the specific biological processes or pathways in which they participate. This deeper understanding aids in elucidating the underlying mechanisms governing biological processes.

For each Gene Ontology (GO) term selected, an in-depth investigation was conducted on the genes that were up- and down-regulated between the two groups. Supplementary Figures S1 and S2 provide detailed visual representations of these findings, illustrating the genes associated with each GO term showing differential expression.

## 4. Discussion

### 4.1. GO Terms in the “na_neg” Phenotype

#### 4.1.1. GOBP_Response_to_Oxygen_Containing_Compound and GOBP_Inflammatory _Response

Any process that leads to a change in the state or activity of a cell of a living organism (in terms of movement, secretion, enzyme production, gene expression, etc.) as a result of a stimulus produces an oxygen-containing compound.

In individuals with ASD, levels of enzymes that scavenge Reactive Oxygen Species (ROS), such as Superoxide Dismutase (SOD), Glutathione Peroxidase (GPx), Catalase, and the antioxidant molecule Glutathione (GSH), are lower compared to healthy controls. Additionally, it has been demonstrated that neuroinflammatory conditions may be present in individuals with ASD, which are commonly associated with and sustained by high levels of ROS [37,38]. Regarding ROS formation, heavy metals (Cd, Pb, arsenic, Hg) have been shown to induce the release of cytotoxic substances, immune responses, neuronal inflammation, and subsequent generation of ROS, thereby causing irreversible damage to the brain development of individuals with ASD [39,40,41].

The association between immune system alterations and ASD has long been hypothesized, but only in recent decades has research focused particularly on this aspect. Studies in individuals with ASD have indicated that immune system dysfunction is often accompanied by a significant inflammatory state [42,43]. Moreover, signs of microglial activation and increased levels of inflammatory cytokines and chemokines, such as Interferon γ (IFN-γ), Interleukin 1-beta (IL-1β), Interleukin 6 (IL-6), Tumor Necrosis Factor (TNF), and Chemokine, Cc Motif, Ligand 2 (CCL2), have been observed in the brain and cerebrospinal fluid of individuals with ASD [44,45]. Furthermore, post-mortem brain samples from individuals with ASD have revealed elevated levels of pro-inflammatory markers [44] and increased microglial activation. Given the current evidence, interventions targeting oxidative stress and inflammation could represent a promising strategy for addressing ASD [37,38].

With reference to Figure 3, among the Gene Ontology (GO) terms associated with up-regulation in the “na_neg” phenotype, particularly those related to inflammatory response and response to oxygen-containing compounds, there are 22 genes that overlap. 

Among these, Caspase Recruitment Domain-Containing Protein 8 (*CARD8*) is involved in the regulation of inflammatory signaling pathways, particularly those involved in immune response and inflammation [46]. Interleukin 18 Receptor Accessory Protein (*IL18RAP*) is a subunit of the interleukin-18 receptor complex, which mediates the effects of interleukin-18, a pro-inflammatory cytokine involved in immune responses [47]. The Neutrophil Cytosolic Factor 1 (*NCF1*) gene is involved in the assembly of the NADPH oxidase complex in neutrophils, which generates ROS [48].

Conversely, among the commonly down-regulated genes in these two pathways are NOD-Like Receptor Family Pyrin Domain-Containing 3 (*NLRP3*), Nuclear Factor Kappa-B Inhibitor Alpha (*NFKBIA*), Nuclear Receptor Subfamily 4 Group A Member 1 (*NR4A1*), and Interleukin 1 beta (*IL1B*). The *NLRP3* gene encodes a protein that is part of the NLRP3 inflammasome complex, which plays a crucial role in regulating the body’s inflammatory response and is implicated in the development of various conditions, including autoimmune disorders and auto-inflammatory diseases [49]. 

*NR4A1* is a gene that codes for a nuclear receptor involved in the regulation of gene expression in response to various cellular signals. It is known to participate in processes such as inflammation and programmed cell death, contributing to the maintenance of cellular homeostasis and the response to environmental stresses [50].

*IL1B* encodes a pro-inflammatory cytokine known as interleukin-1 beta. This cytokine plays a central role in mediating inflammation and immune responses. Its dysregulation has been implicated in various inflammatory and autoimmune diseases, highlighting its significance in maintaining immune balance and homeostasis [51,52].

#### 4.1.2. GOCC_Mitochondrion 

Several studies have identified a 5–80% association between mitochondrial dysfunction and ASD [53,54,55].

In connection with mitochondrial dysfunction and ASD, several studies have reported elevated levels of lactate and pyruvate in the blood and brain, suggesting a malfunction in OXPHOS [53,56,57]. Brain regions with high mitochondrial density, such as dendritic and axon terminals, require efficient mitochondrial function for energy production, calcium homeostasis, and synaptic plasticity [54]. Alterations in bioenergetic or metabolic genes essential for mitochondrial function may lead to abnormalities in brain activity, resulting in cognitive and behavioral abnormalities characteristic of ASD [55]. Moreover, individuals with mitochondrial dysfunction exhibit an accelerated neurodegeneration following infection, suggesting that metabolic decompensation and cytokine toxicity, in the presence of mitochondrial dysfunction, may contribute to autistic regression following infection or vaccination [53].

Examining Figure 3 of Cytoscape, which evaluates the connections between differentially expressed genes and categories of biological processes, molecular functions, or cellular components, we observe that the NADH-Ubiquinone Oxidoreductase Flavoprotein 2 (*NDUFV2*) gene is down-regulated in the cellular component of the mitochondrion. A study by Loser et al. (2023) [58] linked the cell adhesion molecule L1 with the *NDUFV2* gene [58]. The dysfunction of L1 has been associated with abnormalities in neuronal differentiation, migration, survival, axon growth, fasciculation, memory, learning, synaptogenesis, and behavior, all of which can be linked to autism [58]. Loser and colleagues demonstrated that a 70 kDa fragment of L1 (L1-70) is transported into the mitochondria and interacts with mitochondrial proteins, contributing to mitochondrial metabolism, trafficking, synaptic plasticity, motor coordination, neuritogenesis, and cell survival [58].

The DNA Damage Inducible Transcript 4 (*DDIT4*) gene linked to both GOCC_mitochondrion and GOBP_Response_To_Oxygen_Containing_Compound. Luo et al. (2023) [59] found that *DDIT4* expression appears to be involved in ferroptosis in subjects with ASD. *DDIT4* gene protein expression is associated with stress responses such as hypoxia, energetic stress, DNA damage, nutrient depletion, and endoplasmic reticulum stress [59]. Overexpression of DDIT4 can inactivate the PI3K/Akt pathway and promote neuronal ferroptosis, contributing to the onset of autism symptoms [59].

The Solute Carrier Family 25 Member 37 (*SLC25A37*), an iron importer located in the inner mitochondrial membrane, was found to be up-regulated in our studies. Bahado-Singh et al. (2019) [60] analyzed epigenetic modifications, specifically cytosine methylation (CpG), in infants with autism and healthy controls [60]. They identified several genes, including *SLC25A37*, with differential methylation in individuals with autism compared to healthy controls. Methylation affects gene expression, suggesting that the *SLC25A37* gene may be differentially expressed in individuals with autism [60].

#### 4.1.3. GOMF_G_Protein_Coupled_Receptor_Activity

G Protein-Coupled Receptors (*GPCRs*) constitute the largest family of cell-surface receptors that transmit extracellular signals to convergent intracellular signaling pathways and downstream cellular responses, which are frequently dysregulated in ASD. They play pivotal roles in various physiological processes, including sensory perception (such as taste, smell, and vision), neurotransmission, hormone regulation, and immune response [61].

Canonical GPCRs typically possess an intracellular domain composed of the N-terminal and three extracellular loops (EL1–3) connecting seven transmembrane (TM) helices. Ligand binding induces conformational changes in these helices, transmitting activation signals to the intracellular domain, consisting of IL1–3 and a C-terminal region with an eighth helix parallel to the plasma membrane [62].

Recent genome association studies of large ASD cohorts have robustly identified two major convergent neurobiological mechanisms: gene expression regulation or neuronal communication, and localization or plasticity [63,64,65]. Moreover, pharmacological interventions targeting GPCRs hold considerable therapeutic potential for ASD [61].

Furthermore, meta-analyses and transcriptomic data of prefrontal cortex tissue have demonstrated that GPCRs are among the most frequently dysregulated genes in ASD, with approximately 200 GPCRs implicated in the disorder [66,67].

Additionally, Annamneedi et al. (2023) [61] revealed that GPCRs and their downstream signaling pathways are affected by ASD. GPCRs respond to various natural signals, including photons, amino acids, peptides, and large glycosylated proteins.

Referring to Figure 3 of our results, among the differentially expressed genes associated with Protein_Coupled_Receptor_Activity, Inflammatory_Response pathway, and GOCC_Secretory_Vesicle, we highlight the FPR2 gene.

Formyl Peptide Receptors (*FPRs*) belong to a subfamily of class A GPCRs, comprising three isoforms (FPR1, FPR2, and FPR3) in humans [68]. These receptors are functionally expressed on the cell and nuclear membrane of different cell types [69,70,71]. FPRs can recognize various ligands, modulating diverse biological functions such as angiogenesis, metabolism, cell proliferation, and cell death [68,72,73]. Additionally, FPRs can regulate inflammatory responses in numerous pathophysiological processes, including cancer [70,74,75,76], neurodegeneration [77,78,79], and cardiovascular disease [80,81]. FPR2, in particular, is considered the most versatile isoform, capable of recognizing structurally and chemically unrelated ligands [82,83]. *FPR2* can modulate pro-inflammatory responses [68,84] and activate several kinases [85,86,87], signaling proteins [68,73], and NADPH Oxidase-Dependent (NOX) release of ROS [88,89]. Given that our results indicate the overexpression of the gene *FPR2*, this can not only aggravate the inflammatory process in ASD but also potentially play an important role in the general pathogenesis of individuals with ASD.

### 4.2. GO Terms in the “na_pos” Phenotype

#### 4.2.1. GOCC_Neuron_Projection

It is noteworthy to highlight that in this sample of ASD patients, an overall “na_pos” phenotype of the “GOCC_Neuron_projection” pathway was observed. Although the possible explanation underlying this finding is rather complex, it is known that glutamatergic projection neurons generate sophisticated excitatory circuits to integrate and transmit information among different cortical areas, as well as between the neocortex and other regions of both the brain and spinal cord [90]. Additionally, the appropriate development of cortical projection neurons is regulated by certain essential events, such as neural fate determination, proliferation, specification, differentiation, migration, survival, axonogenesis, and synaptogenesis [91]. The generation of correct subtypes and precise connections of projection neurons is imperative not only to support basic cortical functions (such as sensory information integration, motor coordination, and cognition) but also to prevent the onset and progression of neurodevelopmental disorders, including ASD, thus possibly playing a role also for habilitative purposes [92,93]. Indeed, abnormalities in the neocortex, including altered cortical cytoarchitecture and excitatory circuits, are highly associated with neurodevelopmental diseases such as ASD. 

Accordingly, findings from both clinical and preclinical studies demonstrate that defects of neocortical projection neurons associated with mutations of paramount transcriptional factor genes are possible common causes of some neurological diseases, including ASD [94,95]. Moreover, the neocortex is composed of two main types of neurons: excitatory projection neurons and inhibitory interneurons. Abnormalities of interneuron development and function also contribute to the etiology and progress of neurodevelopmental disorders and psychiatric diseases [96]. 

Nonetheless, several questions regarding how and why the establishment of the excitatory–inhibitory balance is so indispensable to maintaining basic neocortical functions and preventing the onset and progression of neurodevelopmental diseases, psychiatric disorders, and even neurodegenerative conditions remain unanswered. In this context, recent evidence in a mouse model showed that embryonic Rbp4-Cre neurons strongly express autism-associated genes, and perturbing these genes interferes with the switch between the two motifs. Hence, pyramidal neurons form active, transient, multi-layered pyramidal-to-pyramidal circuits at the inception of the neocortex, and studying these circuits could yield insights into the etiology of autism [97]. 

Similarly, by using single-nucleus RNA sequencing of cortical tissue from patients with ASD to identify autism-associated transcriptomic changes in specific cell types, it was found that synaptic signaling of upper-layer excitatory neurons and the molecular state of microglia were preferentially affected in autism. Moreover, dysregulation of specific groups of genes in cortico-cortical projection neurons correlated with clinical severity. Overall, these findings suggest that molecular changes in upper-layer cortical circuits are linked to behavioral manifestations of autism [98], thus possibly hypothesizing a pathophysiological role of the GOCC_Neuron_projection “na_pos” phenotype noted in the present sample of ASD patients.

Several studies have identified an implication of the Semaphorin-6A (*SEMA6A*) gene in the development of ASD. This gene, in particular, has been evaluated as being involved in the development of neuronal networks, regulated by intracellular signaling events; furthermore, altered neuronal connectivity is reported in individuals with ASD [99,100,101,102,103]. Menzel et al. (2019), through the use of genetically modified mice, observed how the knockout of *SEMA6A* is relevant for the formation of GABAergic interneurons in several brain areas involved in ASD, such as the primary somatosensory cortical areas, the hippocampus, and the Thalamic Reticular Nucleus (RTN), consequently leading to alterations in excitatory/inhibitory (E/I) balance and neurodevelopmental defects/ASD [99]. Considering that our data show an overexpression of *SEMA6A*, it is reasonable to think that it may have an important role in the phenotype of subjects with ASD, as proposed by the authors mentioned above.

#### 4.2.2. GOBP_Cell_Adhesion 

Another “na_pos” phenotype has been observed: the GOBP_cell_adhesion pathway. Cell adhesion molecules (CAMs) are a group of cell membrane proteins that regulate the cell-extracellular matrix (ECM) and cell–cell interactions [104]. CAMs play a key role in synaptic formation and organization [105,106,107]. Many of them are also involved in synaptic target recognition and subcellular specificity [108,109,110]. Therefore, they are often closely associated with various neurodevelopmental and psychiatric disorders such as autism and other neuropsychiatric disorders [106,111]. The genetic component in ASD is very important, as expressed in previous studies [8,106,111]. 

Candidate genetic variants are often found in genes that code for synaptic cell adhesion molecules, which specify synaptic functions, such as neurexins (NRXNs) and neuroligins (NLGNs). In-Hee Lee et al. (2023) revealed a unique association between specific NLGN1 haplotypes and plasma glutamine levels in individuals with ASD and their family members. They also evidenced a significant correlation between plasma glutamine levels and the severity of restrictive and repetitive behaviors (RRB), a central feature of the disorder. The authors did not find a direct association between *NLGN1* variants and RRB scores, but these data suggest that plasma glutamine levels could potentially serve as an endophenotype, mediating the genetic influence of *NLGN1* on ASD severity on RRB severity [111]. 

Regarding NRXNs, some authors have pointed out that the expression of the *NRXN1* gene may play an important role in depressive disorders [112]. *NRXN1* gene expression appears to be markedly over-expressed in the cohort with autism compared with the control cohort (Supplementary Table S1). These data could suggest further confirmation that this gene could play a decisive role in ASD. 

#### 4.2.3. GOCC_Cell_Surface 

To the GOCC group of the cell surface belong all those genes that are involved in various mechanisms of cell adhesion, interaction with other cells, and cellular signaling pathways. This complexity necessitates a nuanced approach to treatment. Nonetheless, further exploration of these concepts can be found at the following link: https://pantherdb.org/panther/categoryList.do?searchType=basic&fieldName=all&listType=5&fieldValue=CELL%20SURFACE; accessed on 1 January 2020.

Returning to our data, among the differentially expressed genes, we highlight the CD34 antigen (*CD34*) (Figure 3), which is down-regulated, and Interleukin 1 receptor, type I (*IL-1R1*), which is down-regulated.

CD34 is a monomeric cell surface antigen selectively expressed in human hematopoietic progenitor cells. Several neurophysiological alterations have been associated with autism, with immune abnormalities and neural hypoperfusion being considered causative factors. Correlations between altered inflammatory responses and hypoperfusion with symptology have been reported [113]. Cord blood has been successfully used to stimulate angiogenesis in various models of ischemia, with the CD34+ fraction of cord blood possessing the ability to differentiate into endothelial cells [114]. The concentration of this potential endothelial progenitor fraction in cord blood CD34+ cells is approximately ten times higher than in bone marrow CD34+ cells [115]. Numerous studies have confirmed that systemic administration of cord blood cells is sufficient to induce neuroregeneration [116,117]. Given the ability of umbilical cord blood CD34+ cells to induce angiogenesis in areas of cerebral hypoperfusion, some authors have proposed that this type of cell may be useful for the treatment of autism [113]. Thus, the reduced expression of *CD34* in peripheral blood leukocytes that we have highlighted correlates with the data of the authors mentioned above and strengthens the hypothesis of treatment of autistic subjects.

A gene that is up-regulated in GOCC_Cell_Surface is IL1R1, which is a receptor of Interleukin-1 (*IL-1*). *IL-1* plays important roles in many physiological and pathological processes in the central nervous system (CNS) and has been involved in the regulation of sleep and its homeostasis [118], memory consolidation and plasticity [119,120], and neurodegeneration mechanisms [121]. The IL-1R1 mediates IL-1-initiated signaling. *IL-1R1* expression is guided by multiple cell type-specific promoters, allowing control of cell type-specific expression [122]. The results of Liu et al. (2019) [123] show that *IL-1* induces disease behaviors through IL-1R1. Thus, qualitative and quantitative differences mediated by IL-1R1 signaling could explain the different roles of *IL-1* in different diseases. Therefore, the high expression of IL-1R1 highlighted by our data could confirm the pro-inflammatory mechanisms detected in autistic subjects.

#### 4.2.4. GOCC_Secretory_Vesicle 

All cells in the body engage in intercellular communication, which is indispensable for maintaining functions and interactions between different cell populations. This communication primarily occurs through the secretion of molecules within vesicles. Extracellular vesicles (EVs) have been isolated from various body fluids, such as blood, urine, serum, or cerebrospinal fluid (CSF). Several studies have demonstrated that EVs play a significant role in the development of the CNS [124].

Tsilioni and Theoharides (2018) observed a significant increase in extracellular vesicles (EVs) in the serum of children with ASD compared to healthy controls. These vesicles were found to contain mitochondrial DNA (mtDNA) and may stimulate microglia to produce the proinflammatory cytokine IL-1β. This finding suggests that EVs may trigger the proinflammatory response and partially explain the immune dysregulation reported in autistic patients [125].

In our study, the Chemokine, CC Motif, Receptor 3 (*CCR3*) gene appears to be overexpressed (Supplementary Table S1). Studies with isolated human neutrophils have shown that inflammatory mechanisms can be triggered via IFN-γ or TNF-α through CC chemokines, leading to the up-regulation of Chemokine, CC Motif, Receptor 1 (*CCR1*) and CCR3 expression, or via a Chemokine, CC Motif, Receptor 5 (*CCR5*)-mediated mechanism [126,127]. However, the exact role of chemokines and their impact on neutrophils infiltrated at sites of chronic inflammation are poorly understood. Thus, our data on the *CCR3* gene further suggest that inflammatory mechanisms play a role in ASD. Furthermore, our findings align with existing literature regarding the involvement of chemokines in ASD pathology.

## 5. Conclusions

In conclusion, this study conducted a differential expression analysis that identified 733 genes as statistically significant in the autistic groups compared to controls (Supplementary Table S1). Among these, 584 genes were up-regulated, while 149 genes were down-regulated.

Subsequent Gene Set Enrichment Analysis (GSEA) identified the GOCC_Mitochondrion pathway stands out from other pathways, as the differentially expressed genes predominantly belong to this pathway alone. This suggests a significant implication of various mitochondrial functions in ASD. Among the differentially expressed genes, the FPR2 gene (log2FoldChange 2.791) was notably highlighted as belonging to three GO pathways, as shown in Figure 3. Given our preliminary results, albeit based on a limited sample size, the over-expression of the FPR2 gene suggests its potential utility as a biomarker for ASD, particularly in assessing ongoing pro-inflammatory processes. A limitation of the study concerns potential differences in gene expression between PBMCs and neurons/glial cells, where variable gene expression could indicate direct involvement in the development of ASD. Future comprehensive studies are warranted to confirm the validity of this potential biomarker. Another substantial limitation of this study is the small number of samples, which cannot be overcome at present. Additionally, the cohort is composed predominantly of males. 

## Data Availability

Raw data and normalized gene counts are available at ArrayExpress under accession number E-MTAB-13871.

## Supplementary Materials

The following supporting information can be downloaded at: https://www.mdpi.com/article/10.3390/biomedicines12071402/s1, Supplementary Table S1: Differential expression analysis genes as statistically significant (padj ≤ 0.05) between the two groups (autistic vs control); Supplementary Table S2: The table identifies the analysis report of the “Phenotypic Enrichment” section, showing results for gene sets with a negative enrichment score; Supplementary Table S3: The table identifies the analysis report of the “Phenotypic Enrichment” section, showing results for gene sets with a positive enrichment score; Supplementary Figure S1: Expression Heatmap. (A) Heatmap of differentially expressed genes involved in negative regulation of GO term: GOMF_G_Protein_Coupled_Receptor_Activity. (B) Heatmap of differentially expressed genes involved in negative regulation of GO term: GOBP_Inflammatory_Response. (C) Heatmap of differentially expressed genes involved in negative regulation of GO term: GOCC_Mitochondrion. (D) Heatmap of differentially expressed genes involved in negative regulation of GO term: GOBP_Response_To_Oxygen_Containing_Compound. Red indicates that the expression level of the gene is relatively up-regulated, and green indicates that the expression level of the gene is down-regulated; Supplementary Figure S2: Expression Heatmap. (A) Heatmap of differentially expressed genes involved in positive regulation of GO term: GOBP_cell_adhesion. (B) Heatmap of differentially expressed genes involved in negative regulation of GO term: GOCC_Cell_Surface. (C) Heatmap of differentially expressed genes involved in negative regulation of GO term: GOCC_Secretory_Vesicle. (D) Heatmap of differentially expressed genes involved in negative regulation of GO term: GOCC_Neuron_projection. Red indicates that the expression level of the gene is relatively up-regulated, and green indicates that the expression level of the gene is down-regulated.
