# Supplementary material for: Transcriptome Study in Sicilian Patients with Autism Spectrum Disorder
Source: Biomedicines. 2024 Jun 25;12(7):1402. doi: 10.3390/biomedicines12071402 (PMC11274004; doi:10.3390/biomedicines12071402)
Supplement: Supplementary file 1 [file biomedicines-12-01402-s001.zip › Supplementary Table S1.pdf]

Supplemmentary Table S1. The table shows the 733 genes that by differential expression analysis identified them as statistically significant (padj ≤ 0.05) between the two groups (autistic vs. control).

GeneID	log2FoldChange	padj
AP002381.2	4,437	1,73E-16
AC104389.6	9,285	1,45E-12
INKA2	3,175	8,62E-11
TMEM252	8,230	8,75E-10
CSF2RBP1	5,375	1,00E-09
LINC02863	4,423	1,26E-09
AC007271.1	8,081	1,91E-09
AL513320.1	3,696	2,48E-09
LUCAT1	4,337	3,33E-09
KIAA0319	3,658	3,51E-09
AC092145.1	4,184	3,51E-09
SLC45A4	2,777	1,01E-08
AOC3	4,228	1,08E-08
C5AR2	2,499	1,42E-08
AC012291.3	4,254	2,38E-08
AC012645.4	6,380	2,74E-08
STX3	2,371	3,48E-08
SUMO1P1	4,973	3,48E-08
DUSP2	-4,196	3,64E-08
FBXL13	4,204	3,64E-08
ZNF230	1,908	8,54E-08
AL031432.4	3,561	8,70E-08
AC004233.2	4,130	1,67E-07
CEACAM3	4,422	2,08E-07
AC015802.6	4,204	2,16E-07
RPGRIP1	2,445	2,71E-07
DAPK2	2,979	4,32E-07
ADAMTSL4-AS2	3,367	4,47E-07
ADGRE3	3,015	4,79E-07
LINC00921	1,962	6,38E-07
FRAT2	2,832	6,90E-07
HAL	2,371	7,21E-07
SIGLEC8	5,332	1,02E-06
VMP1	1,768	1,17E-06
LINC02218	5,144	1,18E-06
ARHGEF2-AS2	2,500	1,19E-06
LRRK2-DT	2,809	1,19E-06
AC124319.3	3,979	1,39E-06
ZNF200	1,920	2,04E-06
TNFSF14	2,579	2,04E-06

GeneID	log2FoldChange	padj
ARAP3	2,597	2,92E-06
AC092171.4	4,719	2,92E-06
AC007342.2	4,810	2,92E-06
AOC2	3,124	4,38E-06
VNN1	3,492	5,41E-06
TSPEAR	6,788	5,93E-06
LINC00852	-2,414	6,64E-06
TLR6	2,056	6,86E-06
RGS1	-4,897	6,94E-06
HECW2	3,279	8,38E-06
OLIG2	6,179	8,54E-06
IRAG1	2,591	9,18E-06
HSPA6	3,088	9,18E-06
MPZL3	2,129	9,60E-06
ATG16L2	1,920	1,04E-05
MIR29B2CHG	2,632	1,04E-05
MT-TL1	4,549	1,05E-05
EPHB1	2,621	1,15E-05
AC103858.3	2,610	1,16E-05
AATK	3,116	1,30E-05
AC092746.1	4,613	1,30E-05
ISL2	2,831	1,34E-05
TIGD3	3,117	1,34E-05
AP000873.1	2,344	1,54E-05
TRPM6	3,864	1,56E-05
AC132938.5	2,608	1,58E-05
ADAMTSL4-AS1	3,516	1,58E-05
H2BC4	3,198	1,59E-05
CLDN9	4,216	1,59E-05
ZC3H10	1,477	1,64E-05
AC099343.2	3,329	1,65E-05
AC123595.1	5,232	1,90E-05
OR51R1P	7,194	1,90E-05
TRBV5-1	-5,344	2,08E-05
AC027682.7	2,672	2,08E-05
ALOX15	5,059	2,08E-05
AC012645.1	3,841	2,09E-05
VNN2	2,560	2,15E-05
PHF24	6,340	2,25E-05
AL161909.1	-4,478	2,42E-05

GeneID	log2FoldChange	padj
RN7SL600P	3,282	2,95E-05
AC107959.3	3,324	2,95E-05
IL10RB	1,830	3,05E-05
CAPN10-DT	1,939	3,07E-05
CEP63	1,476	3,16E-05
PPP1R3D	2,112	3,35E-05
MPZL1	2,036	3,53E-05
FCGR2A	2,381	3,53E-05
AC015871.8	2,416	3,53E-05
AC012645.2	5,985	3,57E-05
RPL10AP6	-3,112	4,03E-05
ANKUB1	5,325	4,03E-05
LRWD1	1,715	4,30E-05
NUAK2	1,880	4,30E-05
RPL13AP5	-1,888	4,64E-05
NR4A1	-4,271	4,87E-05
TNFRSF10C	4,193	5,28E-05
AC007342.3	2,290	6,07E-05
SNHG22	2,988	6,17E-05
REPS2	2,252	6,86E-05
MIR223HG	2,680	7,26E-05
RN7SKP16	4,304	7,26E-05
Z98886.1	4,785	7,26E-05
CKLF-CMTM1	5,114	7,48E-05
AL109809.1	3,828	8,62E-05
AC012065.3	-3,965	8,76E-05
AC024293.1	-2,445	8,76E-05
H2BC20P	2,206	8,76E-05
LINC02289	4,557	1,02E-04
CEP295NL	3,363	1,03E-04
LUNAR1	3,664	1,03E-04
CCN3	4,114	1,11E-04
TREML2	2,133	1,19E-04
DHX34	2,165	1,23E-04
AC096970.1	4,891	1,23E-04
RN7SL473P	3,677	1,28E-04
GBP6	4,639	1,37E-04
TMEM140	2,833	1,37E-04
PPP1R12B	2,267	1,54E-04
ARHGAP25	1,411	1,55E-04
AL627309.5	2,631	1,55E-04
AC007298.2	3,813	1,55E-04
ALPK1	2,014	1,62E-04
CKLF	1,826	1,74E-04

GeneID	log2FoldChange	padj
YIPF4	1,455	1,84E-04
RAB11FIP1	1,569	1,89E-04
F2RL1	2,758	1,95E-04
IFIT2	3,880	1,98E-04
ID1	-5,039	1,99E-04
ACOX1	1,574	1,99E-04
AC018607.1	3,325	2,04E-04
AP002907.1	3,440	2,25E-04
AC107959.4	4,805	2,25E-04
ZNF252P-AS1	3,179	2,27E-04
CNTNAP3C	8,169	2,27E-04
CSF3R	2,433	2,36E-04
AC138035.1	2,935	2,36E-04
AC022506.2	3,119	2,36E-04
CYP4F3	3,959	2,36E-04
MAK	2,767	2,63E-04
AC020917.4	1,355	2,64E-04
GRAMD1C	2,549	2,83E-04
ST6GALNAC2	2,963	2,83E-04
AC112496.1	3,992	2,83E-04
XPC-AS1	2,961	2,85E-04
RPL7A	-6,129	3,25E-04
AC015871.3	1,495	3,25E-04
AC011676.1	3,667	3,25E-04
CCNJL	3,107	3,44E-04
TRBV29-1	-4,813	3,71E-04
AVIL	2,568	3,78E-04
RPL3P4	-2,275	3,81E-04
CCR3	2,900	3,81E-04
PISD	1,654	3,93E-04
P2RY13	2,216	4,12E-04
C16orf54	1,595	4,20E-04
ABHD5	1,702	4,33E-04
RNF24	2,193	4,40E-04
AC022762.2	2,479	4,50E-04
GK	2,368	4,92E-04
AC011498.4	4,248	5,01E-04
TECPR2	2,020	5,19E-04
TPTEP2	1,961	5,45E-04
H2BC19P	2,176	5,45E-04
AP000692.1	3,126	5,45E-04
AC136475.9	6,174	5,45E-04
CCL3L1	-9,650	5,57E-04
AL162458.1	1,650	5,71E-04

GeneID	log2FoldChange	padj
NR4A2	-4,232	5,79E-04
CXCR2	4,300	5,87E-04
NEAT1	2,113	5,91E-04
AC083862.1	2,366	6,10E-04
RPL21P123	3,807	6,10E-04
NABP1	2,303	6,62E-04
AC123912.2	5,454	6,62E-04
GPR155	1,350	7,44E-04
MTCO3P12	-2,513	7,47E-04
FRAT1	1,726	7,47E-04
ELAPOR1	2,786	7,66E-04
NPL	1,935	8,14E-04
AC099489.1	2,987	8,47E-04
RPS3AP38	3,873	8,47E-04
DPEP3	3,361	8,54E-04
AC025594.2	-3,660	8,92E-04
GOLGA5P1	2,993	8,98E-04
RPS29P12	6,704	8,98E-04
RPL13AP25	-2,680	9,00E-04
TMEM71	1,475	9,00E-04
HOTAIRM1	2,538	9,92E-04
ROPN1L	3,152	1,02E-03
NFKBIA	-4,062	1,06E-03
AC132872.5	2,568	1,10E-03
DUSP4	-3,780	1,11E-03
CEP19	2,911	1,11E-03
NRXN1	5,231	1,11E-03
VNN3	2,742	1,14E-03
AC131009.4	2,514	1,20E-03
NTNG2	2,143	1,21E-03
TMEM154	2,149	1,21E-03
CD83	-4,566	1,22E-03
H2BC18	4,391	1,22E-03
UBR5-AS1	1,681	1,25E-03
STEAP4	3,090	1,25E-03
TUT7	1,438	1,27E-03
AC243772.2	6,300	1,27E-03
AC073342.1	3,953	1,30E-03
TMEM92-AS1	4,078	1,30E-03
GHRL	2,268	1,41E-03
LETM2	1,914	1,41E-03
AL353708.3	2,370	1,41E-03
RAB36	2,080	1,42E-03
AC010201.2	3,671	1,46E-03

GeneID	log2FoldChange	padj
NADK	1,637	1,47E-03
LINC01127	1,996	1,49E-03
AC007342.4	3,443	1,49E-03
AC105046.1	-5,279	1,55E-03
ELF3	-2,802	1,55E-03
AL358473.1	3,858	1,55E-03
AC016737.1	3,079	1,57E-03
AC012651.1	2,193	1,62E-03
KIF27	1,963	1,65E-03
PACSIN2	1,389	1,66E-03
H3-3A-DT	3,413	1,70E-03
FPR2	2,791	1,71E-03
AC092384.3	2,962	1,71E-03
BEST1	2,612	1,75E-03
AL353759.1	2,149	1,90E-03
MX2	1,832	1,94E-03
LINC01270	3,024	1,95E-03
ENTPD1	1,401	1,96E-03
MGAM	4,126	1,97E-03
CARD8	1,391	1,99E-03
BTNL8	4,074	2,04E-03
AL442125.2	4,213	2,04E-03
ST8SIA4	1,445	2,12E-03
AC025031.4	3,265	2,13E-03
CMTM2	4,287	2,15E-03
BASP1-AS1	3,989	2,25E-03
SLC22A1	3,403	2,33E-03
MYBPH	4,841	2,35E-03
LINC00639	5,449	2,36E-03
RASSF2	1,468	2,37E-03
LINC02217	7,244	2,37E-03
DNAJC3-DT	2,189	2,42E-03
TNFAIP3	-3,802	2,43E-03
PRDM5	3,179	2,43E-03
LINC00862	3,549	2,46E-03
PIP4P2	1,834	2,47E-03
CKAP2LP1	5,460	2,47E-03
C10orf105	2,034	2,49E-03
AC011933.2	2,298	2,49E-03
NSUN7	3,063	2,49E-03
Z94721.1	2,775	2,54E-03
ST20	2,384	2,55E-03
MPZ	1,420	2,59E-03
CXCL2	-8,436	2,60E-03

GeneID	log2FoldChange	padj
RGL3	3,156	2,60E-03
LINC02158	2,082	2,67E-03
HSH2D	1,544	2,69E-03
CA15P1	3,016	2,70E-03
AC034236.1	-2,653	2,75E-03
TM6SF1	1,680	2,80E-03
JUN	-3,206	2,89E-03
IKBIP	1,649	2,89E-03
AL359762.3	2,897	2,90E-03
CNTNAP3	3,958	2,90E-03
ST20-MTHFS	2,560	2,94E-03
CDKN1A	-2,669	2,95E-03
FAM157A	2,974	3,01E-03
AC092910.3	1,929	3,05E-03
ZDHHC18	1,762	3,16E-03
AL590867.2	-1,667	3,23E-03
PAN3-AS1	1,668	3,23E-03
KRT5	-3,679	3,26E-03
AC015802.4	2,467	3,32E-03
ZNF132	2,193	3,36E-03
EYS	5,689	3,36E-03
PTX3	-5,100	3,45E-03
GTF2IP4	1,817	3,61E-03
KBTBD6	2,051	3,61E-03
RPH3A	-2,844	3,82E-03
ZNF486	1,690	3,82E-03
MMP25	3,141	3,90E-03
AL021707.1	2,692	3,90E-03
NPM1P27	-1,718	3,92E-03
AC007342.5	2,796	3,92E-03
DUSP5	-1,937	4,02E-03
SLC25A37	3,009	4,06E-03
AC008038.1	-1,469	4,07E-03
AL683842.1	3,184	4,18E-03
IFIT3	3,604	4,18E-03
TSPY26P	-1,861	4,48E-03
PPP1R3B	3,054	4,48E-03
AC127070.4	2,111	4,48E-03
AC021106.1	2,320	4,48E-03
TRBV7-2	-4,950	4,51E-03
THBS1	-3,527	4,51E-03
MPO	-2,272	4,51E-03
NCF1B	1,943	4,52E-03
AC024257.3	2,203	4,53E-03

GeneID	log2FoldChange	padj
SELL	1,536	4,57E-03
LINC01814	2,507	4,59E-03
AC010491.1	2,911	4,59E-03
LINC01001	2,342	4,61E-03
CNIH3	1,628	4,68E-03
RPL23AP42	-1,949	4,70E-03
AL109809.4	2,335	4,74E-03
CFAP58	2,811	4,77E-03
AL354877.1	4,734	4,92E-03
NIBAN1	2,018	5,25E-03
NECAB2	3,782	5,29E-03
AP003498.2	4,634	5,33E-03
CHST15	1,586	5,37E-03
LITAF	1,842	5,37E-03
OTX1	3,776	5,37E-03
AC114811.2	4,658	5,40E-03
AC006330.1	1,892	5,66E-03
CSF2RB	2,502	5,74E-03
CYP4F12	2,750	5,74E-03
ABCG1	1,846	5,82E-03
RNA5SP149	6,392	5,86E-03
AP003108.2	1,424	5,93E-03
HBEGF	-3,267	6,09E-03
AC005280.2	2,732	6,12E-03
CD69	-2,713	6,26E-03
RNU6-920P	4,484	6,31E-03
AC104530.1	1,836	6,41E-03
IFIT5	1,973	6,41E-03
CHI3L1	3,240	6,41E-03
AC011446.1	3,973	6,41E-03
RSAD2	3,857	6,73E-03
RNA5SP99	6,117	6,73E-03
AC027271.1	-4,337	6,83E-03
AC131009.3	2,128	6,83E-03
RERE-AS1	2,297	6,92E-03
ITGAX	1,624	7,05E-03
CMTM1	2,772	7,05E-03
AC017083.1	2,778	7,11E-03
NPIPB11	-1,701	7,24E-03
AC037198.1	-6,536	7,27E-03
SLC16A3	1,836	7,35E-03
CCN2	4,433	7,35E-03
AC008569.2	2,406	7,39E-03
TRIM25	1,598	7,42E-03

GeneID	log2FoldChange	padj
RPL21P16	-2,479	7,49E-03
CASS4	2,107	7,55E-03
DOCK4	2,382	7,55E-03
AL162377.3	2,494	7,55E-03
RNU6-646P	5,363	7,55E-03
MT-TT	-2,386	7,63E-03
AC022211.1	2,934	7,68E-03
OXER1	2,072	7,70E-03
AC010894.4	2,296	7,70E-03
AC005746.1	3,250	7,70E-03
DNAAF1	-8,897	7,72E-03
KAZN	3,524	7,72E-03
AL021707.3	1,897	7,75E-03
ACSS3	2,994	8,00E-03
AC116533.1	-2,120	8,36E-03
NLRP3	-2,806	8,41E-03
JUNB	-2,080	8,41E-03
RPL15P3	-2,036	8,41E-03
MNDA	2,095	8,41E-03
DGAT2	2,441	8,41E-03
TGFA	2,541	8,41E-03
AC092692.1	5,343	8,47E-03
AL031777.2	4,056	8,56E-03
LRRN1	2,505	8,64E-03
SMO	-2,515	8,86E-03
DTX4	1,365	8,86E-03
AREG	-5,571	8,92E-03
MBOAT2	2,040	8,98E-03
AC004584.3	3,417	9,02E-03
AC055764.2	-4,292	9,14E-03
DDIT4	-2,327	9,17E-03
NDUFS5	-1,670	9,30E-03
AC063977.1	2,788	9,48E-03
LRP10	1,483	9,50E-03
LINC02649	1,599	9,96E-03
AC022182.1	2,391	1,00E-02
LINC02555	3,756	1,00E-02
PLPP6	1,349	1,01E-02
UBN1	1,469	1,01E-02
PLIN5	2,998	1,01E-02
MME	3,754	1,01E-02
PRRG4	1,979	1,01E-02
CXCR1	4,204	1,02E-02
AC078962.1	3,506	1,03E-02

GeneID	log2FoldChange	padj
RN7SL1	2,915	1,06E-02
LINC02363	2,678	1,06E-02
STAM-AS1	3,745	1,06E-02
KBTBD7	2,175	1,07E-02
CTBS	1,594	1,07E-02
LINC02362	2,253	1,08E-02
CREB5	2,365	1,08E-02
TPT1P6	-3,692	1,11E-02
IDO1	2,847	1,11E-02
GSEC	3,076	1,13E-02
AC000093.1	4,037	1,13E-02
NHSL2	1,682	1,13E-02
CDC42EP1	-2,359	1,15E-02
AL592429.1	-6,279	1,16E-02
OLR1	-3,637	1,16E-02
PER1	-2,609	1,16E-02
AL592078.1	2,559	1,17E-02
ATF3	-3,262	1,18E-02
AL078604.2	3,233	1,18E-02
IFIT1	3,680	1,19E-02
RNU6-759P	4,536	1,20E-02
ABTB1	2,103	1,20E-02
LFNG	1,491	1,21E-02
PRSS33	3,517	1,21E-02
BEND7	3,133	1,23E-02
AC020911.2	5,579	1,25E-02
AC110769.2	2,999	1,28E-02
CFAP92	2,305	1,32E-02
DENND3	1,520	1,32E-02
SIGLEC5	2,799	1,34E-02
RN7SL648P	3,275	1,36E-02
PEX12	1,710	1,37E-02
HSPA1B	4,965	1,39E-02
RPL5P1	-2,248	1,40E-02
CHKB-CPT1B	1,753	1,41E-02
ERV3-1	1,911	1,42E-02
AL590385.2	2,731	1,42E-02
ANKRD44-IT1	1,946	1,43E-02
LPAR5	1,521	1,43E-02
AL117335.1	2,027	1,43E-02
CES1	-2,070	1,44E-02
AL122020.1	-2,978	1,45E-02
RASGEF1B	-2,815	1,45E-02
RPS14	-1,351	1,45E-02

GeneID	log2FoldChange	padj
TOPORS	1,803	1,45E-02
AL135818.1	1,529	1,48E-02
SLC26A8	2,883	1,48E-02
MYO1F	1,345	1,50E-02
GALNT14	3,359	1,51E-02
AC097634.1	2,558	1,53E-02
LINC01331	5,437	1,53E-02
PTAFR	1,536	1,54E-02
NFE2	2,759	1,54E-02
AC007950.1	5,441	1,55E-02
FOSL1	-4,585	1,59E-02
GPR18	1,352	1,61E-02
AC025031.5	3,383	1,62E-02
ITPKB-IT1	2,250	1,62E-02
RPL13AP7	-1,872	1,63E-02
LBR	1,500	1,67E-02
B3GNT8	2,422	1,67E-02
NFE2L1-DT	2,534	1,69E-02
AC108704.2	2,606	1,70E-02
PRUNE2	3,742	1,70E-02
AC068205.2	2,270	1,71E-02
MCTP2	1,459	1,73E-02
LINC01359	2,527	1,73E-02
TRBV6-5	-4,350	1,75E-02
AC009506.1	2,003	1,75E-02
TAMALIN	-2,833	1,77E-02
CACNB4	2,567	1,77E-02
MSL1	1,324	1,78E-02
HIP1	2,137	1,78E-02
EIF1	-1,350	1,78E-02
ASPRV1	1,914	1,78E-02
RN7SL4P	4,139	1,78E-02
FAM169B	5,665	1,78E-02
AC105339.2	2,299	1,80E-02
ANXA2P2	-2,092	1,84E-02
AC008875.1	2,686	1,84E-02
AC073840.1	-3,951	1,84E-02
NT5DC4	4,031	1,87E-02
SH3BP5L	1,363	1,88E-02
AC016583.1	2,732	1,88E-02
CNOT3	1,358	1,89E-02
PABPN1L	2,871	1,90E-02
AC005329.3	-5,160	1,91E-02
LRFN1	1,554	1,92E-02

GeneID	log2FoldChange	padj
GPR27	1,964	1,93E-02
AC006978.1	-1,578	1,93E-02
AC099524.1	1,473	1,93E-02
AC006033.2	2,089	1,93E-02
AC079316.2	3,506	1,93E-02
Z92544.1	1,681	1,94E-02
SLC19A1	1,946	1,94E-02
LINC02680	2,843	1,95E-02
AC145207.9	3,663	1,97E-02
RPL10P16	-1,498	2,00E-02
SCAT8	2,201	2,02E-02
AC015909.5	2,295	2,03E-02
AC008764.6	3,137	2,03E-02
AP000763.3	1,892	2,04E-02
CACNA1E	4,468	2,04E-02
RPL14P1	-1,634	2,04E-02
KCNJ15	3,355	2,06E-02
AC010335.3	3,735	2,06E-02
AL355802.3	-2,154	2,06E-02
GCA	1,829	2,07E-02
FHDC1	2,885	2,08E-02
CXCL3	-5,600	2,09E-02
MIR6753	1,982	2,12E-02
P2RY14	1,706	2,13E-02
AL360091.2	5,005	2,16E-02
MALAT1	1,500	2,17E-02
DPEP2	1,604	2,17E-02
RASGRP4	1,509	2,22E-02
PLXNC1	1,607	2,23E-02
LINC00683	3,202	2,24E-02
AC006077.2	-2,055	2,24E-02
AC103831.1	5,440	2,25E-02
CCPG1	1,646	2,26E-02
NRN1	3,732	2,26E-02
NCF1	1,974	2,26E-02
IL1R2	2,837	2,26E-02
SIK1B	-2,956	2,28E-02
LINC00674	1,462	2,28E-02
AP001412.1	3,168	2,28E-02
AL928646.1	5,435	2,29E-02
PANX2	2,518	2,31E-02
RPL7P9	-1,699	2,31E-02
NRBF2	1,778	2,34E-02
AC008764.8	1,832	2,34E-02

GeneID	log2FoldChange	padj
AC044849.1	-3,538	2,40E-02
FAM174A	1,701	2,42E-02
UBE2FP1	-2,718	2,42E-02
CD34	-2,186	2,44E-02
DAAM2	3,648	2,44E-02
SULT1B1	1,838	2,48E-02
AC018690.1	-2,127	2,50E-02
EEPD1	1,380	2,50E-02
CNGA4	2,964	2,51E-02
NFE4	4,787	2,52E-02
IGKV3-15	-3,202	2,54E-02
MADCAM1	1,913	2,54E-02
PEAK3	2,826	2,54E-02
H1-6	4,531	2,54E-02
H4C5	3,498	2,55E-02
AC090616.6	-1,521	2,57E-02
AC002091.2	1,561	2,57E-02
AC025682.1	1,850	2,57E-02
MIRLET7BHG	2,291	2,57E-02
AC005363.2	5,514	2,57E-02
RN7SL5P	5,334	2,57E-02
ZNF239	1,901	2,59E-02
G0S2	-5,995	2,64E-02
SEPTIN5	2,876	2,64E-02
AL139099.2	5,191	2,64E-02
IL1R1	2,288	2,66E-02
IL18RAP	1,721	2,67E-02
AC068790.5	4,350	2,67E-02
ADRA2B	-8,626	2,67E-02
AC009948.3	2,990	2,67E-02
AC004951.1	1,426	2,68E-02
C3orf86	3,240	2,68E-02
PPP1R15A	-2,719	2,68E-02
AC096921.2	-1,529	2,68E-02
AP003717.1	-1,381	2,68E-02
NFAM1	1,577	2,68E-02
LRRC4	2,610	2,69E-02
AL139220.2	3,540	2,69E-02
LRP1	-1,598	2,69E-02
AL137792.1	3,956	2,69E-02
RNU2-6P	4,001	2,69E-02
SP2-AS1	3,501	2,72E-02
RNASEL	1,941	2,74E-02
LINC01303	3,547	2,74E-02

GeneID	log2FoldChange	padj
ETV3L	-4,578	2,76E-02
AL442128.2	1,936	2,76E-02
MIR6813	2,387	2,76E-02
SLC5A10	-2,625	2,78E-02
ZFYVE16	1,444	2,79E-02
AC100778.2	2,689	2,80E-02
RPS7P3	-3,163	2,81E-02
AC008770.3	1,587	2,81E-02
BATF2	3,080	2,82E-02
COL6A1	-1,905	2,83E-02
PLEKHG3	1,395	2,83E-02
CDHR5	-4,073	2,84E-02
AC004951.2	1,734	2,85E-02
OIT3	4,959	2,85E-02
LINC01506	3,085	2,85E-02
BX640514.2	2,333	2,92E-02
FAM157B	2,452	2,92E-02
WLS	1,596	2,92E-02
OR52K3P	1,797	2,93E-02
ADGRG3	2,844	2,93E-02
AL592114.1	-2,890	2,94E-02
AC108463.3	2,865	2,95E-02
RBKS	-2,262	2,96E-02
AC215522.2	2,276	2,96E-02
TMEM160	-1,450	2,97E-02
AL355309.1	-3,308	2,97E-02
MINDY1	1,866	3,00E-02
DRAIC	2,941	3,02E-02
FCGR3B	4,267	3,03E-02
SEC14L2	-2,462	3,03E-02
C5orf67	3,331	3,09E-02
SPART-AS1	-4,498	3,09E-02
HNRNPA1P52	2,547	3,09E-02
MN1	-3,227	3,14E-02
DDX10P1	3,672	3,15E-02
SLC30A6-DT	-3,496	3,15E-02
SORL1	1,607	3,15E-02
U73169.1	1,803	3,15E-02
SIRPB1	1,574	3,18E-02
PADI2	2,090	3,18E-02
AC068790.2	6,325	3,20E-02
AC079250.1	-1,724	3,21E-02
NYNRIN	-2,475	3,22E-02
RPL4P7	2,629	3,22E-02

GeneID	log2FoldChange	padj
AC130456.3	4,183	3,24E-02
JAML	1,939	3,25E-02
AC009065.5	1,912	3,27E-02
KCNK17	-3,842	3,27E-02
AC103591.3	-5,620	3,28E-02
RUNX3	-1,381	3,28E-02
MSRB1	2,134	3,28E-02
RPS2P5	-1,461	3,30E-02
ZNF879	1,453	3,30E-02
AC108448.2	-3,273	3,33E-02
RPL17P10	3,362	3,34E-02
U6	3,102	3,36E-02
PELO	1,336	3,37E-02
AC074132.1	3,460	3,42E-02
AC095055.1	-3,534	3,45E-02
AC007569.1	2,171	3,45E-02
AL357078.2	3,766	3,45E-02
H4C4	3,115	3,46E-02
AC137630.3	-3,176	3,46E-02
LCNL1	-2,900	3,46E-02
IGKV1-27	-3,592	3,47E-02
ATP2B1-AS1	-2,666	3,47E-02
IL6R	1,506	3,47E-02
SEMA6A	5,722	3,47E-02
PLAU	-3,618	3,48E-02
MIR320E	2,235	3,48E-02
AC016831.4	2,942	3,48E-02
RPS7P1	-1,979	3,50E-02
WNT7A	-1,653	3,51E-02
IFITM2	2,518	3,51E-02
PPIAP29	2,561	3,51E-02
H2AC15	2,936	3,51E-02
AL732372.2	1,854	3,57E-02
AC008737.1	3,135	3,59E-02
NCBP2-AS1	-3,978	3,59E-02
SNX29P1	3,143	3,59E-02
LINC01002	1,888	3,64E-02
ZNF674	1,424	3,66E-02
EEF1A1P9	-1,623	3,67E-02
RTEL1P1	2,897	3,68E-02
AC104695.2	2,649	3,71E-02
SNAI1	-4,343	3,72E-02
CACNA1D	1,836	3,72E-02
BASP1	2,098	3,72E-02

GeneID	log2FoldChange	padj
AL136531.2	3,898	3,75E-02
AC020765.5	3,439	3,75E-02
CCL20	-8,024	3,75E-02
LINC01936	5,900	3,77E-02
NT5C2	1,342	3,80E-02
RPS27AP5	-2,180	3,82E-02
AP000254.2	-1,628	3,82E-02
SEC14L1	1,669	3,83E-02
AL160408.2	-2,273	3,84E-02
BEAN1-AS1	2,576	3,84E-02
AC010883.3	2,416	3,87E-02
AC087284.1	2,412	3,88E-02
CLEC11A	-1,832	3,90E-02
RUBCNL	1,568	3,90E-02
NCF4	2,543	3,90E-02
XPO6	1,516	3,94E-02
AC007496.3	3,510	3,96E-02
H2BC6	1,904	3,97E-02
PBLD	1,371	4,00E-02
LINC00654	1,806	4,04E-02
AC005306.1	2,041	4,10E-02
AC019254.1	3,656	4,10E-02
IGKV3-20	-2,863	4,11E-02
AVPI1	-2,129	4,13E-02
AC093525.7	4,289	4,13E-02
FPR1	1,731	4,14E-02
AL160272.1	2,155	4,16E-02
AC106037.2	2,493	4,16E-02
DUSP1	-2,404	4,24E-02
AC023908.3	2,039	4,24E-02
LINC01762	3,013	4,27E-02
AL731563.3	1,501	4,29E-02
AL118508.3	2,538	4,29E-02
C15orf48	-5,744	4,29E-02
AL138895.1	1,948	4,29E-02
DHRS9	2,730	4,30E-02
KIAA0825	1,837	4,30E-02
SMAD6	-3,315	4,32E-02
FAM8A1	1,550	4,33E-02
RAB3D	1,705	4,33E-02
IGHD	4,259	4,35E-02
AC120114.3	1,379	4,35E-02
AC073575.2	-1,975	4,37E-02
SLC12A7	-1,559	4,38E-02

GeneID	log2FoldChange	padj
ABCA7	1,390	4,38E-02
ZBTB18	1,526	4,38E-02
IL5RA	2,557	4,38E-02
CFAP58-DT	2,993	4,38E-02
RN7SL517P	3,462	4,38E-02
CYP4F10P	5,296	4,38E-02
CXCL8	-4,978	4,39E-02
TCP11L2	1,531	4,42E-02
KCNE3	1,489	4,48E-02
TNFRSF1A	1,436	4,49E-02
JCAD	6,373	4,52E-02
AC002075.2	-2,195	4,52E-02
ST6GALNAC6	1,611	4,52E-02
IL1B	-4,934	4,52E-02
AL391832.2	2,262	4,52E-02
IGHEP2	-2,342	4,55E-02
LPAR2	1,770	4,58E-02
NDUFV2	-2,434	4,58E-02
PTPRN2	1,800	4,58E-02
EPN2	1,323	4,60E-02
NDEL1	1,605	4,61E-02
CACNG8	2,713	4,63E-02
AC130895.1	4,769	4,64E-02
CHMP4BP1	-3,014	4,77E-02
CNRIP1	-2,639	4,78E-02
EFCAB1	5,169	4,79E-02
AL139042.1	5,759	4,80E-02
AC104806.2	-2,336	4,81E-02
LINC01004	1,340	4,81E-02
TAFA2	1,779	4,84E-02
APOL2	1,514	4,86E-02
MXD1	2,118	4,86E-02
ZNF772	2,180	4,86E-02
GRM2	-1,679	4,98E-02
SAP30L-AS1	1,489	4,98E-02
ACTBP8	5,665	4,99E-02
