# Supplementary material for: Transcriptome Study in Sicilian Patients with Autism Spectrum Disorder
Source: Biomedicines. 2024 Jun 25;12(7):1402. doi: 10.3390/biomedicines12071402 (PMC11274004; doi:10.3390/biomedicines12071402)
Supplement: Supplementary file 1 [file biomedicines-12-01402-s001.zip › Supplementary Table S2.pdf]

Supplementary TableS2. The table identifies the analysis report of the "Phenotypic Enrichment" section, showing results for gene sets with a negative enrichment score.

NAME	SIZE	ES	NES	NOM p-val	FDR q-val	FWER p-val
GOBP_RESPONSE_TO_OXYGEN_CONTAINING_COMPOUND	49	-0,4993532	-3,0845022	0	0	0
GOBP_INFLAMMATORY_RESPONSE	46	-0,4255462	-2,5944126	0	0,001710923	0,004
GOCC_MITOCHONDRION	25	-0,24654184	-1,2293924	0,19433199	0,36080542	1
GOMF_G_PROTEIN_COUPLED_RECEPTOR_ACTIVITY	20	-0,15693864	-0,6906134	0,8484849	0,92077434	1

Legend: SIZE, number of genes in the gene set after filtering out those genes not in the expression dataset. **ES**, Enrichment score for the gene set; that is, the degree to which this gene set is overrepresented at the top or bottom of the ranked list of genes in the expression dataset. **NES**, Normalized enrichment score; that is, the enrichment score for the gene set after it has been normalized across analyzed gene sets. **NOM p-val**, Nominal p value; that is, the statistical significance of the enrichment score. The nominal p value is not adjusted for gene set size or multiple hypothesis testing; therefore, it is of limited use in comparing gene sets. **FDR q-val**, False discovery rate; that is, the estimated probability that the normalized enrichment score represents a false positive finding. **FWER p-val**, Familywise-error rate; that is, a more conservatively estimated probability that the normalized enrichment score represents a false positive finding. Because the goal of GSEA is to generate hypotheses, the GSEA team recommends focusing on the FDR statistic.
