# Supplementary material for: Transcriptome Study in Sicilian Patients with Autism Spectrum Disorder
Source: Biomedicines. 2024 Jun 25;12(7):1402. doi: 10.3390/biomedicines12071402 (PMC11274004; doi:10.3390/biomedicines12071402)
Supplement: Supplementary file 1 [file biomedicines-12-01402-s001.zip › Supplementary Table S3.pdf]

Supplementary Table S3. The table identifies the analysis report of the "Phenotypic Enrichment" section, showing results for gene sets with a positive enrichment score.

NAME	SIZE	ES	NES	NOM p-val	FDR q-val	FWER p-val
GOCC_NEURON_PROJECTION	24	0,25783876	1,0240525	0,42876345	1	1
GOBP_CELL_ADHESION	49	0,20888579	1,0114892	0,45925927	1	1
GOCC_CELL_SURFACE	36	0,20183247	0,9063886	0,5934641	1	1
GOCC_SECRETORY_VESICLE	40	0,17196667	0,79191697	0,7449495	1	1

Legend: SIZE, number of genes in the gene set after filtering out those genes not in the expression dataset. **ES**, Enrichment score for the gene set; that is, the degree to which this gene set is overrepresented at the top or bottom of the ranked list of genes in the expression dataset. **NES**, Normalized enrichment score; that is, the enrichment score for the gene set after it has been normalized across analyzed gene sets. **NOM p-val**, Nominal p value; that is, the statistical significance of the enrichment score. The nominal p value is not adjusted for gene set size or multiple hypothesis testing; therefore, it is of limited use in comparing gene sets. **FDR q-val**, False discovery rate; that is, the estimated probability that the normalized enrichment score represents a false positive finding. **FWER p-val**, Familywise-error rate; that is, a more conservatively estimated probability that the normalized enrichment score represents a false positive finding. Because the goal of GSEA is to generate hypotheses, the GSEA team recommends focusing on the FDR statistic.
