# Supplementary material for: Transcriptome Study in Sicilian Patients with Autism Spectrum Disorder
Source: Biomedicines. 2024 Jun 25;12(7):1402. doi: 10.3390/biomedicines12071402 (PMC11274004; doi:10.3390/biomedicines12071402)

Supplementary Figure S1. Expression Heatmap. (**A**) Heatmap of differentially expressed genes involved in negative regulation of GO term: GOMF_G_Protein_Coupled_Receptor_Activity. (**B**) Heatmap of differentially expressed genes involved in negative regulation of GO term: GOBP_Inflammatory_Response. (**C**) Heatmap of differentially expressed genes involved in negative regulation of GO term: GOCC_Mitochondrion. (**D**) Heatmap of differentially expressed genes involved in negative regulation of GO term: GOBP_Response_To_Oxygen_Containing_Compound. Red indicates that the expression level of the gene is relatively up-regulated, and green indicates that the expression level of the gene is down-regulated.

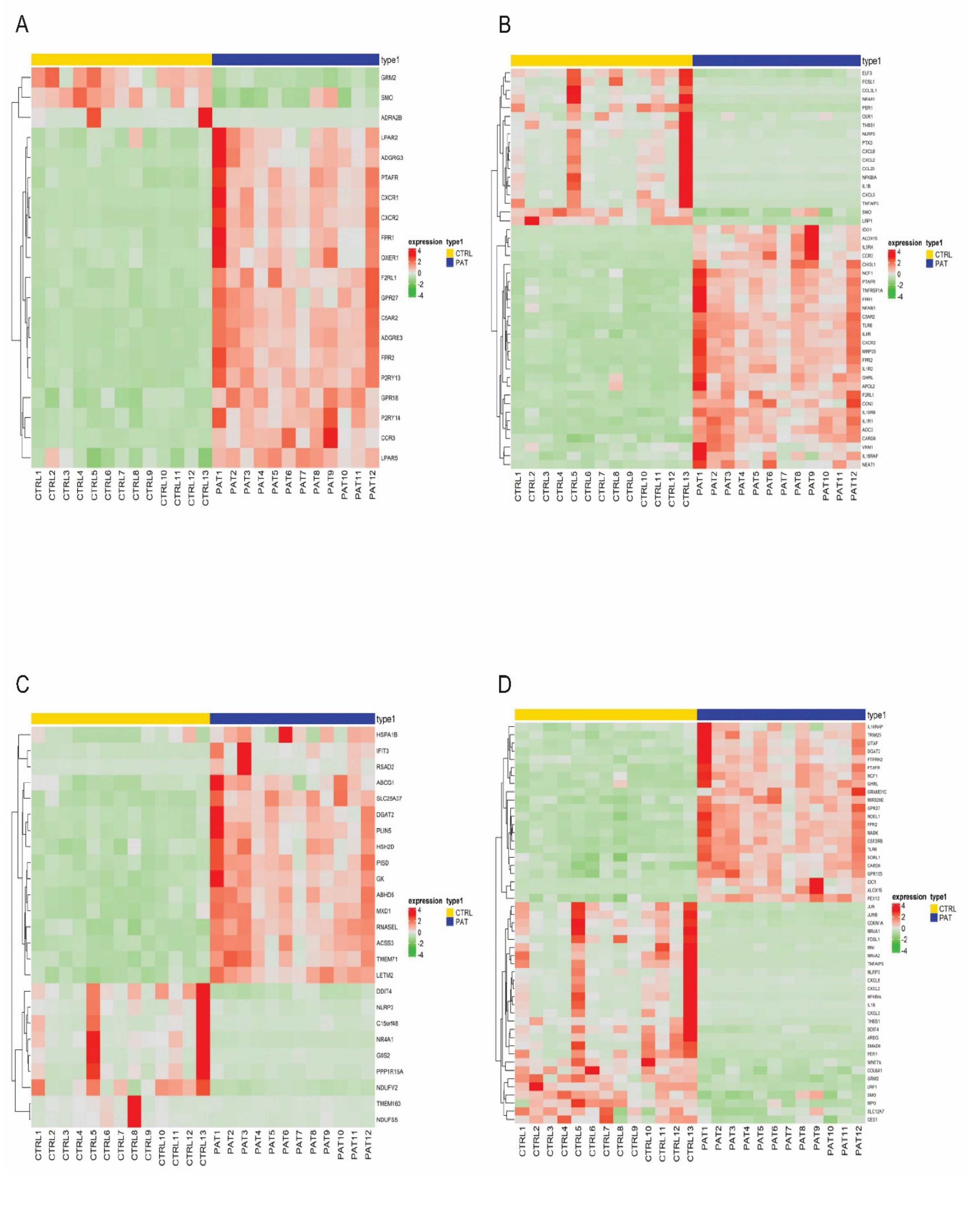

Supplement: Supplementary file 1 [file Supplementary Figure S1.pdf › Supplementary Figure S1.pdf]
